# Endoscopic removal of an extraluminal gastrointestinal stromal tumor recurrence located on the surgical stapler line at gastroesophageal junction

**DOI:** 10.1055/a-2113-9985

**Published:** 2023-07-13

**Authors:** Thomas Thomaidis, Anyi Y. Xiang, Orestis Lyros, Ilias Athanasiadis, George Kallimanis, Dimitris Papaioannou, Ping-Hong Zhou

**Affiliations:** 1 Second Department of Gastroenterology, Hygeia Hospital, Athens, Greece; 2 First Medical Department, Johannes-Gutenberg University of Mainz, Mainz, Germany; 3 Endoscopy Center and Endoscopy Research Institute, Zhongshan Hospital, Fudan University, Shanghai, China; 4 First Department of General Surgery, Hygeia Hospital, Athens, Greece; 5 Department of Oncology, Mitera Hospital, Athens, Greece; 6 Department of Pathology, Hygeia Hospital, Athens, Greece

A 58-year-old man was diagnosed 4 years ago with a 9-cm gastrointestinal stromal tumor (GIST) of the gastric fundus. Under neoadjuvant treatment with imatinib, the GIST was substantially reduced in size and subsequently removed via laparoscopic fundus resection. Despite adjuvant treatment, a recurrence appeared 3 years later at the surgical site on the staple line ( Fig. 1 a ). Owing to a secondary mutation on exon 18 of c-kit, treatment with ripretinib was initiated 1 , under which the GIST reduced in size, from 3 cm to 2.3 cm. Subsequently, surgical removal was proposed but the patient preferred an endoscopic approach.

**Fig. 1:**
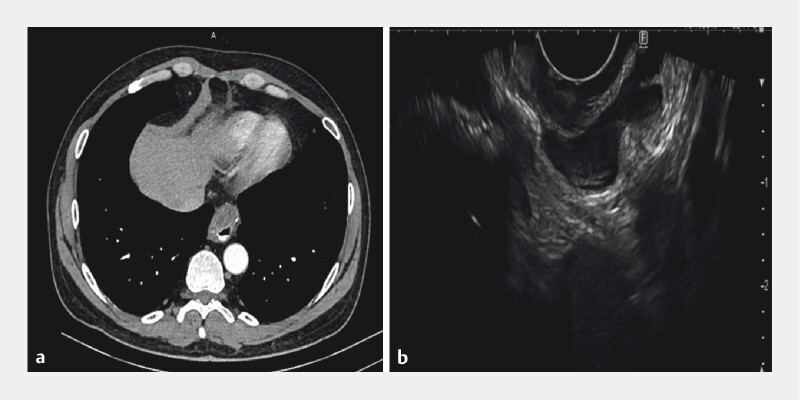
Location of the recurrent gastrointestinal stromal tumor. **a** Computed tomography showing the recurrent lesion located at the surgical site on the staple line. **b** Endoscopic ultrasound identifying the extraluminal growth pattern of the tumor.

Therefore, we performed submucosal tunneling endoscopic resection (STER). The tumor was located extraluminally and could not be identified during endoscopy ( Fig. 1 b ). Via endoscopic ultrasound, we marked the superficial mucosa with a small biopsy. Subsequently, we created an opening to the submucosal space 5 cm proximal to the tumor in the esophagus. We used a therapeutic scope (GIF-1TH190, Olympus, Tokyo, Japan) to create a submucosal tunnel until we reached the staple line. We carefully removed the surgical clips while protecting the integrity of the mucosal layer until we located the tumor ( Fig. 2 , Video 1 ). Finally, the GIST was resected and removed through the tunnel ( Fig. 3 ). The mucosal defect at the tunnel entry point and the biopsy site were closed with clips. The patient received pre-emptive antibiotic treatment and was discharged 3 days later uneventfully. The final esophagogram confirmed mucosal integrity ( Fig. 4 ). Histology revealed complete removal of a 2.1-cm GIST with minimal mitotic rate ( Fig. 5 ).

**Fig. 2:**
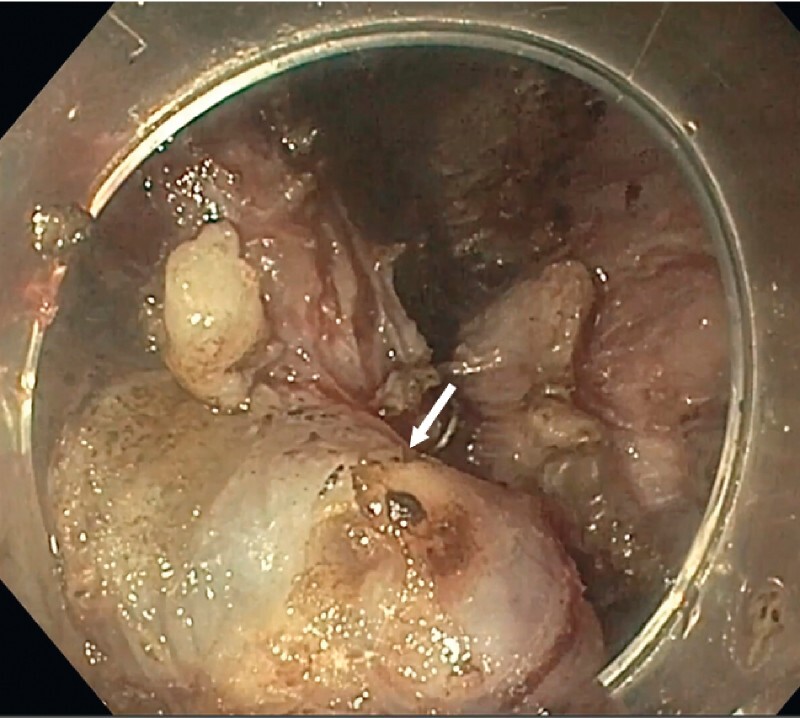
The tumor was barely visible in the tunnel. After dissecting the fibrous tissue, the surface of the tumor was identified (arrow).

**Video 1** Submucosal tunneling endoscopic resection for a recurrent gastrointestinal stromal tumor at the surgical anastomosis.


**Fig. 3:**
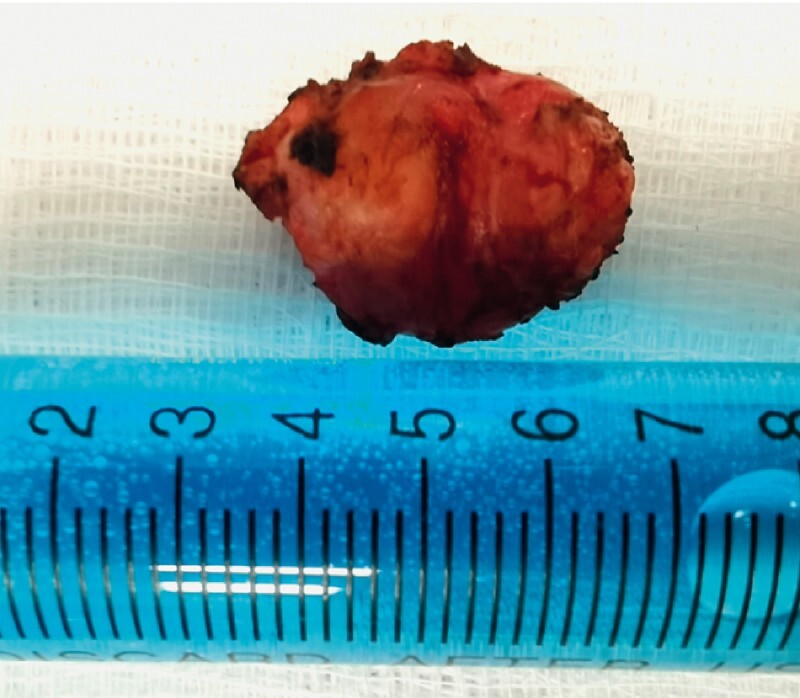
Specimen of the extracted lesion. A 3-cm roundish soft tissue mass was removed.

**Fig. 4:**
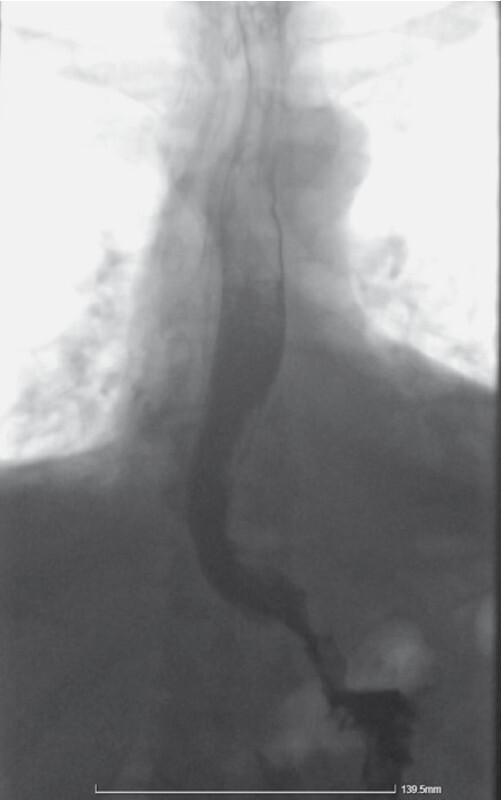
Post-procedural esophagogram confirming mucosal integrity.

**Fig. 5:**
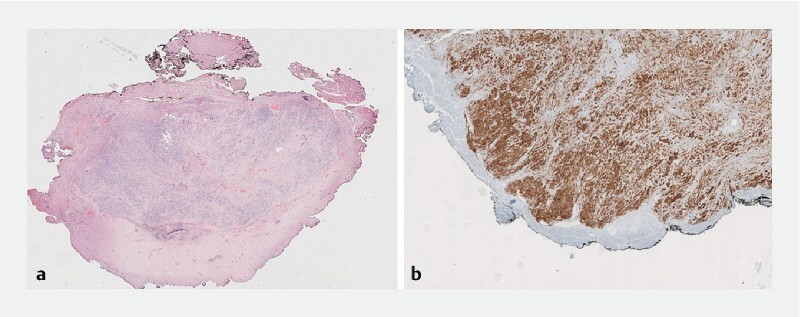
Final histopathology. **a** Panoramic view of the specimen stained with hematoxylin and eosin. **b** The edge of the specimen stained with discovered on GIST 1 (DOG-1) stain.

Endoscopic resection has been suggested as an alternative to surgical excision for GISTs, provided that complete removal is technically possible 2 3 4 . Yet, as far as we know, there are no previous reports describing the application of endoscopic resection on recurrent GISTs. In this case, we achieved complete resection of the recurrent lesion that had persisted even after ripretinib treatment. Moreover, the tumor was located extraluminally at the previous surgical site on the staple line, where severe fibrosis is anticipated. The STER procedure was of high technical difficulty due to the extremely fibrotic surrounding tissue along with the presence of surgical clips, which had to be removed without injuring the superficial mucosa.

To our knowledge, this is the first case of complete endoscopic removal of GIST recurrence at the surgical site on the gastroesophageal junction in a patient under treatment with a tyrosine kinase inhibitor.

Endoscopy_UCTN_Code_TTT_1AQ_2AD

## Notes

Endoscopy E-Videos

https://eref.thieme.de/e-videos
*E-Videos* is an open access online section of the journal *Endoscopy* , reporting on interesting cases and new techniques in gastroenterological endoscopy. All papers include a high-quality video and are published with a Creative Commons CC-BY license. Endoscopy E-Videos qualify for HINARI discounts and waivers and eligibility is automatically checked during the submission process. We grant 100% waivers to articles whose corresponding authors are based in Group A countries and 50% waivers to those who are based in Group B countries as classified by Research4Life (see: https://www.research4life.org/access/eligibility/ ). This section has its own submission website at https://mc.manuscriptcentral.com/e-videos
