# Correction: Endoscopic removal of an extraluminal gastrointestinal stromal tumor recurrence located on the surgical stapler line at gastroesophageal junction

**DOI:** 10.1055/a-2161-3703

**Published:** 2023-09-07

**Authors:** Thomas Thomaidis, An-Yi Xiang, Orestis Lyros, Ilias Athanasiadis, George Kallimanis, Dimitris Papaioannou, Ping-Hong Zhou

**Affiliations:** 1 Second Department of Gastroenterology, Hygeia Hospital, Athens, Greece; 2 First Medical Department, Johannes-Gutenberg University of Mainz, Mainz, Germany; 3 Endoscopy Center and Endoscopy Research Institute, Zhongshan Hospital, Fudan University, Shanghai, China; 4 First Department of General Surgery, Hygeia Hospital, Athens, Greece; 5 Department of Oncology, Mitera Hospital, Athens, Greece; 6 Department of Pathology, Hygeia Hospital, Athens, Greece

**Correction**

**Correction: Endoscopic removal of an extraluminal gastrointestinal stromal tumor recurrence located on the surgical stapler line at gastroesophageal junction** Thomaidis T, Xiang A-Y, Lyros O et al. Endoscopic removal of an extraluminal gastrointestinal stromal tumor recurrence located on the surgical stapler line at gastroesophageal junction. Endoscopy 2023; 55: E896–E897, DOI: 10.1055/a-2113-9985 In the above-mentioned article, the name of the author An-Yi Xiang has been corrected. This was corrected in the online version on August 31, 2023.

